# Stepwise Management of Refractory Ventricular Arrhythmias in Desmoplakin Cardiomyopathy: A Case Report

**DOI:** 10.7759/cureus.99789

**Published:** 2025-12-21

**Authors:** Grace Ibitamuno, Erica D Wittwer, Ruben J Crespo-Diaz, Mauricio A Villavicencio, Brendan T Wanta

**Affiliations:** 1 Anesthesiology and Perioperative Medicine, Mayo Clinic, Rochester, USA; 2 Cardiovascular Medicine, Mayo Clinic, Rochester, USA; 3 Cardiovascular Surgery, Mayo Clinic, Rochester, USA

**Keywords:** arrhythmogenicity, cardiac transplantation, cardiomyopathy, desmoplakin, mechanical circulatory support, stellate ganglion block

## Abstract

Desmoplakin cardiomyopathy is a rare, genetic condition that often leads to dangerous arrhythmias, ventricular scarring, and eventual heart failure. Here, the case of a 50-year-old woman suffering from both ventricular storm and biventricular heart failure is discussed. Her ventricular tachycardia proved resistant to several antiarrhythmic medications, which made it difficult to maintain proper cardiac output. To stabilize her condition temporarily, a stellate ganglion block was performed until she could undergo successful orthotopic heart transplantation. When antiarrhythmic drugs are unsuitable or fail to control electrical storms, stellate ganglion blockade presents a valid alternative. For patients with desmoplakin cardiomyopathy, especially when combined with mechanical circulatory support, this approach can serve as an effective bridge to cardiac transplant.

## Introduction

Desmoplakin (DSP) cardiomyopathy is a form of arrhythmogenic cardiomyopathy (ACM) characterized by potentially fatal arrhythmias and episodic cardiac myocyte injury leading to ventricular fibrosis [1,2]. The prevalence of ACMs has been estimated at 0.02% - 0.05%, with 60% of all cases thought to be associated with pathogenic mutations. Specifically, 3% - 20% of ACM cases are attributed to DSP mutations, though the overall prevalence of ACM cases is likely underestimated due to variability across populations [3-5]. Guideline-recommended treatment of ACMs includes pharmacologic therapy, genetic testing for risk stratification and counseling, secondary prevention of cardiac arrest via implantable cardioverter-defibrillator (ICD) or catheter ablation, and avoidance of strenuous activities [6].

Desmoplakinopathies are associated with a hereditary mutation in the desmosomal DSP protein responsible for cell-cell adhesion, causing alterations in the action potentials of myocytes, and together with extensive subendocardial ventricular fibrosis, predisposing to life-threatening arrhythmias and cardiac arrest [7-9]. Phenotypically, desmoplakinopathies have a female predominance, with typical features including palmoplantar keratosis and woolly hair [7]. DSP cardiomyopathy carries a particularly high arrhythmic risk compared to other ACM subtypes, with sustained ventricular arrhythmia rates as high as 3.9-5.9% per year [7].

DSP mutations lead to a high predisposition to ventricular arrhythmias, and placement of an ICD is recommended as secondary prevention, though not without significant risk of increased morbidity from ICD shocks [2]. The management of incessant ventricular tachycardia in DSP cardiomyopathy, particularly in the setting of concomitant heart failure requiring inotropic support, can be clinically challenging where competing physiologic interests are at play. This report presents a case of severe DSP cardiomyopathy that led to incessant ventricular tachycardia and severe biventricular heart failure, leading to the urgent need for cardiac transplantation. The management of incessant ventricular tachycardia with concomitant severe biventricular heart failure is highlighted. This report underscores an algorithmic approach to intervention in highly refractory arrhythmias associated with a complex genetic cardiomyopathy and adds insight to the frequency of use and efficacy of stellate ganglion blockade in the case of very refractory ventricular tachycardia.

## Case presentation

Written informed consent was obtained from the patient for publication of this report. A 50-year-old female with medical comorbidities, including Crohn’s disease, hyperlipidemia, and a 25-pack-year smoking history, presented to the emergency department after an out-of-hospital cardiac arrest, wherein return of spontaneous circulation (ROSC) was achieved after two automated external defibrillator shocks. History revealed a previous episode of syncope six months prior to presentation, which resolved without intervention.

**Figure 1:**
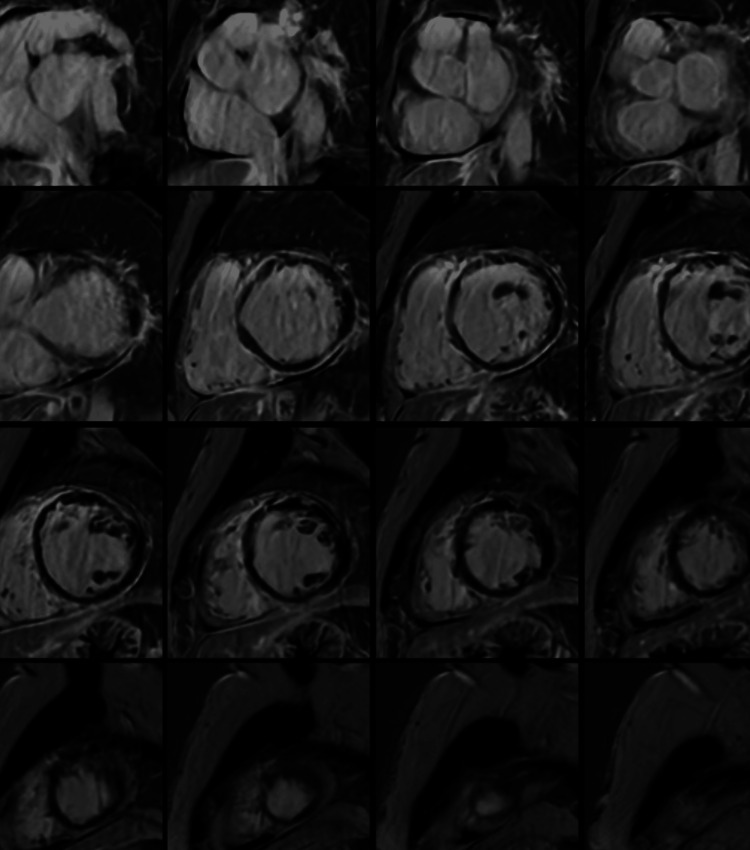
Gadolinium-enhanced cardiac MRI showing diffuse subepicardial and pericardial enhancement in addition to a severely dilated left ventricle with increased trabeculation and a mildly dilated right ventricle

Her workup was significant for a transesophageal echocardiogram showing reduced left ventricular ejection fraction at 20-25%, severely dilated left ventricle, and regional wall motion abnormalities. Left and right catheterization showed no evidence of epicardial coronary obstruction. Cardiac magnetic resonance imaging showed diffuse subepicardial to mid-myocardial late gadolinium enhancement and pericardial enhancement, which is characteristic of desmoplakinopathy (Figure 1). A single-chamber rate-responsive ICD was placed as secondary prevention. The patient presented four days after discharge again, with symptomatic ventricular ectopy, and was placed on oral amiodarone. She underwent a fluorodeoxyglucose-positron emission tomography scan that ruled out sarcoidosis.

Eight weeks after her initial presentation, she was admitted to the hospital secondary to multiple ICD shocks (40 joules) in the setting of symptomatic ventricular tachycardia (up to 214 beats/min that would not convert with antitachycardia pacing therapies), with plans for epicardial ablation to prevent ICD storm. Troponin T was notably elevated (63 ng/L), consistent with myocardial injury. In the catheterization laboratory, substernal epicardial mapping was completed, but ablation was aborted due to inadvertent anterior right ventricular laceration. She was resuscitated with blood products and rushed to the operating room, where she underwent sternotomy and primary closure, then was transferred to the cardiac surgical intensive care unit (CVICU). At this time, genetic testing had revealed desmoplakinopathy with a mutation in p.R2166* (c.6496C>T).

In the CVICU, she required norepinephrine and epinephrine in the setting of cardiogenic shock but continued to experience ventricular ectopy; hence, she was started on amiodarone and lidocaine infusions. The lidocaine infusion was paused after four days of therapy due to supratherapeutic serum levels and limited efficacy. She remained a candidate for extracorporeal membrane oxygenation as a bridge to needed cardiac transplantation.

Four days after primary closure, she continued to experience incessant ventricular tachycardia. A left-sided stellate ganglion block was performed with 10 mL of 0.25% bupivacaine under dynamic ultrasound guidance as a sympathectomy, with continued amiodarone infusion in the background. Epinephrine was replaced with vasopressin to support end-organ perfusion and eliminate exogenous catecholamine administration. Following the stellate ganglion block and the elimination of epinephrine, there were no additional ICD shocks and a noticeably shorter duration in runs of ventricular tachycardia. However, five days later, milrinone was started due to a suboptimal cardiac index (CI) of less than 2 L/min/m^2^. She experienced several runs of prolonged ventricular tachycardia, suffered cardiac arrest, and required chest compressions to achieve ROSC. Five days after the initial block, a repeat left-sided stellate ganglion blockade was again performed with bupivacaine, and she was listed as status 1 for cardiac transplantation due to progressive heart failure and refractory ventricular tachycardia. An intra-aortic balloon pump was placed to support her blood pressure, while frequent runs of ventricular tachycardia continued, and remained in place for two days. Repeat left-sided stellate ganglion blockade was performed once more as a salvage measure, as the patient had progressively more frequent and longer runs of ventricular tachycardia (seven days after the initial block). She ultimately underwent successful orthotopic cardiac transplantation one day after salvage stellate ganglion blockade without significant complications in the postoperative period and was discharged 15 days post-transplant.

## Discussion

**Figure 2:**
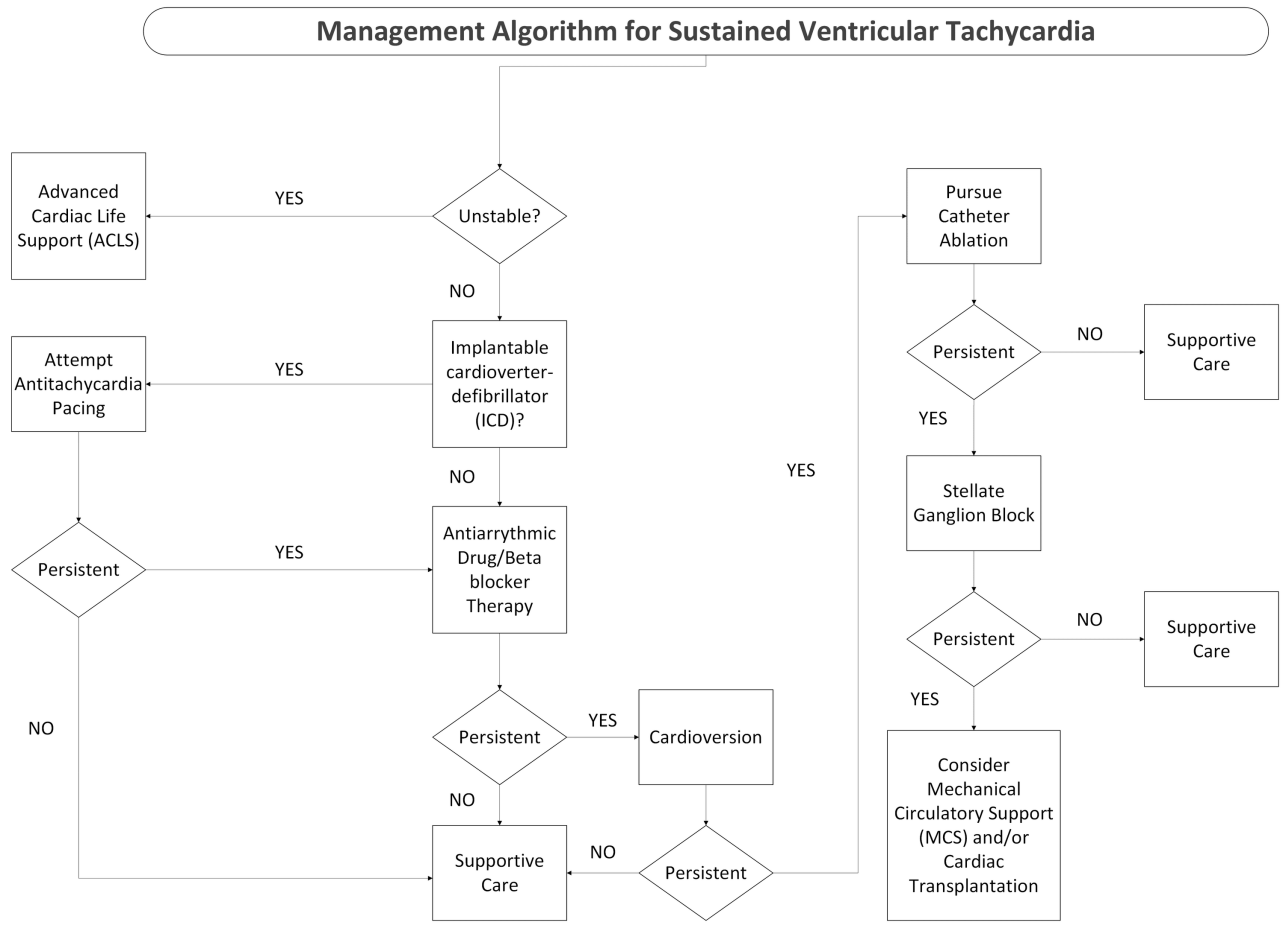
Algorithmic approach to sustained ventricular tachycardia Image was created by the authors based on the 2017 AHA/ACC/HRS Guideline for Management of Patients with Ventricular Arrhythmias and the Prevention of Sudden Cardiac Death [10] and the authors' clinical approach.

Patients with DSP cardiomyopathies may present in an overlap of overt and progressive ACM disease phases, with co-occurring arrhythmogenicity and biventricular heart failure. Managing concomitant arrhythmias and heart failure inherently involves competing physiologic interests. The goal of forward flow accomplished by the addition of inotropic support (catecholaminergic agents/phosphodiesterase inhibitors/calcium chloride) often comes with increased arrhythmogenicity, which in turn leads to worsening cardiac output due to impaired ventricular filling and decreased synchrony of contractions. Multiple treatment approaches are often necessary, as shown in Figure 2 [10].

Antiarrhythmic drugs are typically the easiest and safest first-line therapy in the setting of recurrent ventricular tachycardia, but it is imperative to consider that antiarrhythmics, such as amiodarone, are associated with severe primary graft dysfunction and vasoplegia after heart transplantation [11]. When antiarrhythmic drugs are inappropriate or ineffective, stellate ganglion blockade is a viable, feasible, and safe option for the management of incessant ventricular arrhythmias, remaining so amid biventricular heart failure [12,13]. Its use in managing ventricular sympathetic activity has received a class IIB rating by the American Heart Association and American College of Cardiology in their most recent guideline for management of patients with ventricular arrhythmias [10]. In this case, a left-sided stellate ganglion block was utilized for its predominant effect on arrhythmogenicity. Left-sided stellate ganglion blocks are thought to mitigate ventricular arrhythmias by reducing cardiac sympathetic tone, which can be a key driver for refractory ventricular arrhythmias [13]. Right-sided blockade can also be considered, but it is typically more associated with rate control [14]. Right-sided block was not pursued in this case due to periods of sinus bradycardia (query amiodarone-induced) and the need for heart rate to augment cardiac output in the setting of low stroke volume. Interestingly, although the immediate effects of bupivacaine are short-lived (e.g., Horner’s syndrome) based on the drug’s pharmacokinetics, the decrease in arrhythmogenicity has been shown to last longer than 72 hours in the majority of patients with electrical storm [15]. Although not fully elucidated, this prolonged effect may be because the local anesthetic allows the ganglion to reset, thus reducing the sympathetic surge.

Stellate ganglion blockade may suppress arrhythmias, providing time for other therapeutic options to be instituted, and appears to be successful, albeit short-lived, in the patient at hand. Given this patient’s desmoplakinopathy and the additional factor of cardiac anatomical disturbance and primary repair, treatment options toward the reduction of morbidity and the prevention of imminent mortality were required, namely, mechanical circulatory support (intra-aortic balloon pump) and, ultimately, cardiac transplantation. While extracorporeal membrane oxygenation (ECMO) support was not needed for this patient due to a rapidly available organ, venoarterial ECMO is a feasible option as a bridge to cardiac transplantation in patients with incessant arrhythmias listed for cardiac transplantation.

All inotropes are arrhythmogenic, hence detailing a delicate balance in patients with cardiogenic shock. Epinephrine and milrinone were particularly poorly tolerated in this patient, causing worsening ventricular storm, and in the case of milrinone, cardiac arrest. Interestingly, the effect was not dose-dependent, and worsening dysrhythmia was seen even at low doses. Due to incessant ventricular tachycardia, the patient was weaned off all inotropic support and switched to vasopressin to support end-organ perfusion, though it provides no augmentation to cardiac output. Serial lactate levels were measured in the setting of low CI to ensure adequate oxygen delivery, and serial physical exams were completed to monitor the mesentery. Antiarrhythmic drugs were continued, and a repeat attempt at catheter ablation was considered, though ultimately deferred due to the high-risk nature of the procedure and high likelihood of recurrence in the setting of a progressive disease process. Additional consideration was given to sotalol, but it was deemed to be less efficacious than amiodarone by the electrophysiology team, and amiodarone was therefore continued.

This report is limited by the fact that it is a single-patient case with a rare pathology, which restricts the generalizability of its findings. Furthermore, the simultaneous use of multiple therapeutic interventions makes it difficult to attribute specific outcomes to any one treatment, preventing clear inference of causality for each intervention’s effectiveness.

## Conclusions

In cases of recurrent ventricular tachycardia with a concomitant low cardiac output state, a multifaceted and patient-tailored approach to hemodynamics is necessary. When inotropic support cannot be tolerated, antiarrhythmic therapy followed by catheter ablation is considered the first-line treatment. Stellate ganglion blockade can be used as a temporizing measure or a bridge to recovery. When ablation is not an option, mechanical circulatory support can be considered to either support high-risk ablation or as a bridge to decision or transplantation. Devices such as intra-aortic balloon pumps, percutaneous left ventricular assist devices (LVADs), and extracorporeal membrane oxygenation (ECMO) are all viable options depending on the patient’s degree of cardiac dysfunction. A multidisciplinary team-based approach is often necessary in these challenging scenarios.
